# Wearable devices may aid the recognition of fluctuation-related pain in Parkinson’s disease—An exploratory, cross-sectional analysis of two prospective observational studies

**DOI:** 10.1371/journal.pone.0316563

**Published:** 2025-01-14

**Authors:** Katarina Rukavina, Juliet Staunton, Pavlos Zinzalias, Magdalena Krbot Skoric, Kit Wu, Kirsty Bannister, Alexandra Rizos, K. Ray Chaudhuri

**Affiliations:** 1 Institute of Psychiatry, Psychology & Neuroscience at King’s College London, London, United Kingdom; 2 Parkinson’s Foundation Centre of Excellence, King’s College Hospital NHS Foundation Trust, London, United Kingdom; 3 Department of Neurology, University Hospital Center Zagreb, Zagreb, Croatia; Dokkyo Medical University: Dokkyo Ika Daigaku, JAPAN

## Abstract

Fluctuation-related pain (FRP) affects more than one third of people with Parkinson’s disease (PwP, PD) and has a harmful effect on health-related quality of life (HRQoL), but often remains under-reported by patients and neglected by clinicians. The National Institute for Health and Care Excellence (NICE) recommends The Parkinson KinetiGraph^TM^ (the PKG^TM^) for remote monitoring of motor symptoms. We investigated potential links between the PKG^TM^-obtained parameters and clinical rating scores for FRP in PwP in an exploratory, cross-sectional analysis of two prospective studies: “*The Non-motor International Longitudinal*, *Real-Life Study in PD—NILS*” and “*An observational-based registry of baseline PKG™ in PD—PKGReg*”. 63 PwP (41.3% female; age: 64.24±9.88 years; disease duration, DD: 6.83±5.63 years; Hoehn and Yahr Stage, H&Y: 2 (1–4); Levodopa Equivalent Daily Dose 535 (0–3230) mg) were included. PwP with FRP (n = 23) had longer DD (8.88 (1.29–19.05) vs. 3.16 (0.34–28.92), *p* = 0.001), higher severity of motor symptoms (H&Y 3 (1–4) vs. 2 (1–4), *p* = 0.015; SCOPA Motor total score 21.35±10.19 vs. 13.65±8.99, *p* = 0.003), more dyskinesia (SCOPA Motor Item 18 ≥1 60.9% vs. 7.5%, *p*<0.001), and worse HRQoL (PDQ-8 Total Score 10.74±5.98 vs. 6.78±5.13, *p* = 0.007) then PwP without FRP (n = 40). In the multivariate logistic regression, after the adjustment for DD, H&Y and SCOPA-Motor total score, the presence of FRP was significantly associated with the PKG^TM^-derived Fluctuation-dyskinesia score (Exp (B) = 1.305, 95% CI for Exp (B) 1.012–1.683, *p* = 0.040) and the Bradykinesia score (Exp (B) = 0.917, 95% CI for Exp (B) 0.842–0.999, *p* = 0.048). The PKG^TM^ system may potentially advance the way we screen for, assess, and treat FRP in clinical practice.

## Introduction

Parkinson’s disease (PD) affects over 8.5 million people globally and is, owning to its sharply rising prevalence, emerging as a leading source of disability [1–4]. In addition to its defining motor features (*bradykinesia*—slowness of movement and decrement in amplitude or speed when movements continue; *rigidity*—velocity-independent resistance to passive movement and/or *rest tremor*—4- to 6-Hz tremor in the fully resting limb), an array of nonmotor symptoms (NMS) may be present in affected individuals [5, 6]. Over two thirds of People with Parkinson’s (PwP) live with chronic PD-related pain, with weighty consequences on health-related quality of life (HRQoL) [7]. PD-related pain is ranked highly among the most bothersome symptoms of PD, may be severe enough to overshadow motor symptoms and substantially restricts everyday activities [8–10].

PD-related pain is multifaceted and, according to the International Association for the Study of Pain (IASP) mechanistic descriptors, may be classified as nociceptive, neuropathic, and/or nociplastic [11, 12]. In addition, using a PD-specific, validated clinical assessment tool–the King’s Parkinson’s Disease Pain Scale (KPPS), it can be sub-classified based on its clinical presentation into musculoskeletal, chronic, fluctuation-related, nocturnal, oro-facial, pain related to discoloration; oedema or swelling and radicular pain [13]. When selecting the most appropriate analgesic strategy for PD-related pain, correct recognition of its distinctive subtypes is key [14, 15]. Fluctuation-related pain (FRP) is pain associated with levodopa-induced on/off fluctuations. Defined by the KPPS as either “dyskinetic pain”, “OFF period dystonia”, or “generalized OFF period pain”, FRP is, with prevalence of 33.2–41.9%, the second most prevalent PD-pain subtype (after the musculoskeletal pain), and significantly restricts HRQoL (as measured using EQ-5D-3L and PDQ-8 questionnaires in a cross-sectional study with 178 PwP and 83 healthy controls) [7, 10, 13]. Of note, while oral levodopa substitution remains the gold standard for symptomatic treatment of PD, its chronic use is associated with clinically heterogeneous response fluctuations–both motor (including early morning OFF, wearing OFF, delayed ON, dose failure/no ON or random/unpredictable ON–OFF and dyskinesia) and nonmotor fluctuations (NMF; fluctuations of neuropsychiatric, autonomic, and sensory symptoms), as comprehensively reviewed elsewhere [16–18]. FRP arises as an integral part of NMF and may accompany levodopa-induced motor fluctuations: in a study with 100 PwP, in one third of patients, pain worsened in severity (measured on the 101-point numeric rating scale) in the motor defined OFF state [18].

Despite its high prevalence and harmful effect on HRQoL, PD-related chronic pain often remains under-reported by patients and neglected by clinicians [19, 20]. For example, in a study using Non-motor Symptoms Questionnaire (NMSQuest) [21], over 40% of PwP did not declare their lived experience of persistent pain at their clinical appointment [20]. As a result, many PwP, particularly those from ethnic minority background, are left without adequate pain relief [22].

Past years have seen considerable advances in the field of digital health technologies (DHT), including smartphone apps, wearable sensors and platforms that provide remote healthcare (telehealth); the recent COVID-19 pandemic has further fuelled the demand for their use and facilitated their implementation in clinical practice [23]. Increasingly, DHTs are being employed for assessment, management, and prevention of pain [24]. In PD, five measurement systems are currently endorsed by the National Institute for Health and Care Excellence (NICE) in the United Kingdom (UK) for remote monitoring of motor symptoms: KinesiaU, Kinesia360, PD Monitor, STAT-ON, and the Parkinson KinetiGraph^TM^ (the PKG^TM^; Global Kinetic Corporation, Melbourne, Australia); the latter being backed by the most robust evidence [25]. In previous studies, distinctive PKG^TM^ -based outcome measures reliably identified the presence of motor fluctuations and were associated with a range of NMS (including excessive daytime sleepiness and night-time sleep disturbances, impulsive-compulsive behaviour disorder, gastrointestinal symptoms, sexual dysfunction, mood, cognition, and perceptual problems) [26–31]. However, to date, a potential link between the PKG^TM^-obtained parameters and clinical ratings for pain in PwP has not been explored. Here, we hypothesize that the PKG^TM^-scores may be associated with the presence of FRP in PwP.

## Methods

### Study design

This is an exploratory, retrospective, cross-sectional, one-point-in-time analysis of two prospective, observational studies:

- “*The Non-motor International Longitudinal*, *Real-Life Study in PD—NILS*”: a combined natural history and treatment effect-based study, where NMS are evaluated on an annual basis to explore their evolvement over time, their response to the conventional treatment and their impact on the health-related quality of life (HRQoL)
  and
- “*An observational-based registry of baseline Parkinson’s KinetiGraph™ in Parkinson’s disease—PKGReg*”: an international, multicentre observational-based registry coupling objective scores provided by the PKG^TM^ system with standard, routinely used clinical scales and questionnaires.

Individuals with PD receiving their clinical care at the Movement Disorders Outpatient Clinics at the Parkinson’s Foundation Centre of Excellence at King’s College Hospital NHS Foundation Trust in London, UK were assessed for eligibility during their routine clinical visits. Participants of all genders and all age groups, with diagnosis of PD based on the UK Brain Bank Criteria and within 5 years since the PD onset, were offered the participation in both studies, while those with atypical parkinsonism, concomitant severe disease (conditions interfering with PD assessments) and those unable to give informed consent were not eligible, as described before [26, 32, 33]. Importantly, to avoid any bias caused by pain arising from other, non-PD-related, pain-promoting conditions, individuals with acute or chronic pain better accounted for by any aetiology other than PD (as determined by their treating neurologists, based on detailed medical history and thorough neurological and general clinical examination, including distinctive diagnostic procedures where appropriate), were not included, as reported previously [34]. This analysis was conducted on the sample consisting of participants recruited in the period between 1st July 2020 and 31st December 2022. The anonymized data was accessed on 7^th^ January 2023.

### Assessments

Data on participants’ demographic (age and gender) and disease-related characteristics (disease duration, current medication) were obtained through a structured interview and noted from the NILS and PKGReg databases. Levodopa Equivalent Daily Dose (LEDD) was calculated for each participant [35].

Clinical assessments were performed during routine clinical visits and included:

*King’s Parkinson’s Disease Pain Scale (KPPS)*–this 14-item, 7-domain, rater-completed scale is the first scale developed and validated specifically for the assessment of PD-related pain and is widely used in observational and interventional (pharmacological and non-pharmacological) trials worldwide [34, 36–38]. KPPS classifies PD-related pain into 7 different subtypes (domains) and rates their severity (0–3) and frequency (0–4) over the past month. For each item, severity and frequency are multiplied, resulting in a sub-score of 0 to 12, with a total possible score (a sum of all item scores) ranging from 0 to 168 [13]. Here, the presence of FRP was defined as KPPS Domain 3 score ≥ 1.

*Hoehn and Yahr (H&Y) stage–*a 5-point scale for grading of the degree of patients’ disability and general functional level into 5 categories: unilateral disease (H&Y Stage I), bilateral disease with intact balance (H&Y Stage II), the presence of postural instability (H&Y Stage III), loss of physical independence (H&Y Stage IV) and being wheelchair- or bed-bound (H&Y Stage V) [39].

*Short Parkinson’s Evaluation Scale (SPES)/SCales for Outcomes in Parkinson’s disease–Motor Function (SCOPA-Motor)*—this 21-item, four-response options (ranging from 0 (normal) to 3 (severe)), rater-based scale consists of 3 sections: Motor Evaluation (Section A), Activities of Daily Living (Section B) and Motor Complications (Section C) [40].

*The Parkinson’s Disease Questionnaire—Short Form (PDQ 8)* is an 8-item, patient completed questionnaire. This PD-specific measure of self-perceived health status covers eight dimensions of ill-health rated with scores from 0 (“never”) to 4 (“always” or “cannot do at all”) [41].

### PKG^TM^ evaluation

The PKG^TM^ provides objective, continuous and automated remote (home-based) assessment of motor symptoms in PD. This wrist-worn (on the primarily affected wrist of an individual with PD) watch-like device contains a 3-axis accelerometer, set to record acceleration with sampling rate of 50 samples per second. Data is recorded continuously over a 6-day period, downloaded, quantified, and correlated with the timings of medication intake using propriety software to generate a detailed report. The report includes scores on bradykinesia, dyskinesia, tremor, and immobility, as well as medication adherence, motor fluctuations and periods when the watch is not worn. Once it becomes available, the healthcare professional is automatically alerted [42–44].

**Fig 1:**
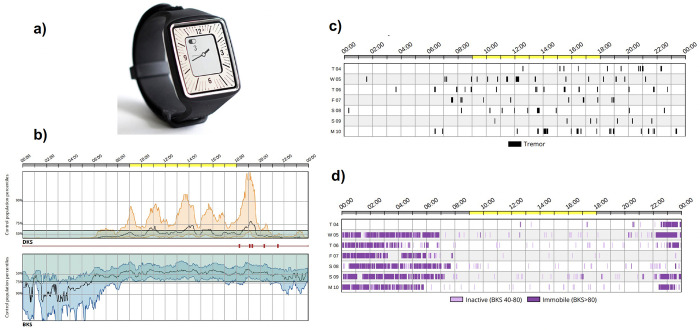
a) The Parkinson’s KinetiGraph™ (The PKG^TM^, Global Kinetics Corporation) wrist-worn device. b) Chart presenting bradykinesia and dyskinesia by the time of the day, in comparison to the scores obtained in healthy controls (bold grey horizontal lines represent median scores in healthy controls) c) A raster plot presenting periods of tremor and d) immobility by the time of the day. Each black dot represents a 2-minute epoch. Reprinted from https://pkgcare.com/wp-content/uploads/2021/09/Whitepaper_The-Long-Term-Leader-in-Parkinsons-Disease-Enhancing-your-Clinical-Trial-Assessment-MM-96.pdf under a CC BY license, with permission, original copyright 2021.

The algorithm recognizes bradykinesia as epochs of movements with a lower acceleration and lower amplitude, and with longer intervals between the movements. Dyskinesia is recognized as reduced intervals between the movements, while the amplitude and the acceleration are both normal. The severity levels for bradykinesia and dyskinesia are defined based on the average 50^th^, 75^th^ and 90^th^ percentiles of the bradykinesia and dyskinesia recorded in healthyindividuals. The PKG^TM^ is programmed to vibrate when a medication is due and allows patients to confirm the actual medication intake by placing their thumb on a sensor zone [42] Fig 1.

The following PKG^TM^ outcome measures were analyzed:

*Bradykinesia Score (BKS)*–the median value of BKS for each 2-minute epoch over the period from 05:00 to 21:00 over six consecutive days *Dyskinesia Score (DKS)*—the median value of DKS for each 2-minute epoch over the period from 05:00 to 21:00 over six consecutive days [26].

*Fluctuation and Dyskinesia Score (FDS)*–a summary score combining the variations in both BKS and DKS. FDS distinguishes between fluctuating and non-fluctuating patients and quantifies the degree of symptom variability [31]. In previous studies, the FDS threshold that separates fluctuators from non fluctuators has been set at 7.7 [31].

*Percentage of Time with Tremor (PTT)–*summary score of tremor periods [45].

and

*Percentage of Time Immobile (PTI)—*periods of immobility defined as the BKS score >80, possibly indicative of periods of daytime sleep or somnolence [28].

### Statistical analysis

All data were analysed using the SPSS Statistics software, version 26.0 (IBM SPSS for Mac, Armonk, NY, USA, IBM Corp.). One-sample Kolmogorov–Smirnov test was applied to test for the normality of the data distribution and the descriptive statistics provided. Group comparisons were carried out using the Independent Samples T-Test, Mann-Whitney test, or the Chi-squared test, as appropriate. The relationship between the PKG^TM^ parameters (BKS, DKS, FDS, PTI and PTT) and other possible clinical cofounders (DD, H&Y, LEDD and SCOPA-Motor total score) with the presence of FRP was investigated in a set of univariate logistic regression models. Statistically significant predictors of FRP presence identified in univariate logistic regression were included in the multivariate logistic regression analysis. Statistical significance was set as p ≤ .05.

### Ethical approvals

Both studies were authorized by local ethic committees: NILS study by the South East London Research Ethics Service (REC reference number 10/H0808/141) and PKGReg study by London Riverside Research Ethics Committee (REC reference number 17/LO/1010) and adopted as national studies by the National Institute of Health Research in the United Kingdom (UK; NILS—UK National Institute for Health Research Clinical Research Network (UKCRN) No.10084; PKGReg—UKCRN No. 215965). All data were handled in compliance with the Data Protection Act 2018 (UK Public General Acts, 2018 2c). Written informed consent was obtained from all participants pior to the inclusion.

## Results

Our analysis included 63 PwP (41.3% women; age: 64.24 ± 9.88 years; DD: 6.83 ± 5.63 years; H&Y: 2 (1–4); LEDD: 535 (0–3230) mg, Table 1). The participants’ clinical characteristics and PKG outcome scores are shown in the Table 1.

**Table 1:** Patients‘ demographic and clinical characteristics and the Parkinson’s KinetiGraph™ (PKG^TM^, Global Kinetics Corporation) scores.

*N = 63*	
Age (Years; Mean±SD)	64.24±9.88
Sex (Women: n, %)	26 (41.3%)
Hoehn & Yahr stage (Median, Range)	2 (1–4)
SCOPA-Motor scale, total score (Mean±SD)	16.46±10.08
SCOPA-Motor -Presence of dyskinesia (n, %)	17 (27%)
SCOPA-Motor -Presence of “off” periods (n, %)	34 (54%)
Disease duration (Years; Mean±SD)	6.83±5.63
LEDD (mg; Median, Range/ Mean±SD)	535 (0–3230) 682.47±596.46
KPPS total score (Mean±SD)	17.11±16.19
Pain medication consume (n, %)	28 (44.4%) NSAIDs: 24 (38.1%) Opioids: 7 (11.1%) Cortisone injections: 1 (1.6%)
PDQ-8 total score (Mean±SD)	8.22±5.74
BKS (Mean±SD)	29.96±7.66
DKS (Median, Range/ Mean±SD)	1.1 (0.1–24.5) / 2.44±3.87
FDS (Mean±SD)	8.63±3.88
PTI (Mean±SD)	10.56±8.44
PTT (Median, Range/ Mean±SD)	5.2 (0.1–42.6) / 10.12±12.33

*KPPS*–King’s Parkinson’s Disease Pain Scale, *SD*–standard deviation, *LEDD*–Levodopa Equivalent Daily Dose, *PDQ-8* –Parkinson’s Disease Questionnaire–Short Form, *SCOPA-Motor*–Scales for Outcomes in Parkinson’s Disease–Motor Function, *BKS*—Bradykinesia Score, *DKS*—Dyskinesia Score, *FDS–*Fluctuation and Dyskinesia Score, *PTT*–Percentage of Time with Tremor, *PTI*–Percentage of Time Immobile

In this cohort, musculoskeletal pain (KPPS Domain 1 score ≥ 1) was the most prevalent pain subtype, present in 77.8% of the participants, followed by FRP (KPPS Domain 3 score ≥ 1, 36.5%), nocturnal pain (KPPS Domain 4 score ≥ 1, 33.3%) and radicular pain (KPPS Domain 7 score ≥ 1, 27%), while other subtypes of PD-related pain (chronic (KPPS Domain 2 score ≥ 1, 22.2%), discoloration; oedema/swelling (KPPS Domain 6 score ≥ 1, 12.7%) and oro-facial pain (KPPS Domain 5 score ≥ 1, 7.9%)) were less prevalent.

Participants who declared presence of FRP (n = 23) had significantly longer disease duration and significantly higher total burden of pain (KPPS total score) then participants without FRP (n = 40). Median H&Y stage, motor symptoms burden (SCOPA-Motor total score), as well as prevalence of dyskinesia (SCOPA-Motor Item 18 ≥ 1), were higher in the group with FRP, and their HRQoL (PDQ-8 total score) was worse, compared with participants without FRP (n = 40), as shown in the Table 2. The two groups did not differ regarding age, gender or LEDD.

**Table 2:** Comparison of the demographic and clinical characteristics between the groups of the participants with and without fluctuation-related pain.

	Fluctuation-related pain + *n = 23*	Fluctuation-related pain—*n = 40*	*p*
Age (Years; Mean±SD)	64.44±10.34	64.12±9.73	0.908
Gender (Female: n, %)	10 (43.5%)	16 (40.0%)	0.797
**Hoehn&Yahr stage (Median, Range)**	**3 (1–4)**	**2 (1–4)**	0.015*
**SCOPA-Motor scale, total score (Mean±SD)**	**21.35±10.19**	**13.65±8.99**	0.003*
**SCOPA-Motor, presence of dyskinesia (Item 18) (n, %)**	**14 (60.9%)**	**3 (7.5%)**	<0.001*
SCOPA-Motor, presence of OFF periods (Item 20) (n, %)	16 (69.6%)	18 (45%)	0.071
**Disease duration (Years; Median, Range)**	**8.88 (1.29–19.05)**	**3.16 (0.34–28.92)**	0.001*
LEDD (mg; Mean±SD)	850.06±711.98	586.11±503.53	0.091
**PDQ-8 total score (Mean±SD)**	**10.74±5.98**	**6.78±5.13**	0.007*
**KPPS total** **(Mean±SD)**	**26.13±19.61**	**11.93±11.15**	0.003*

Based on the normality of the distribution, the data is shown as mean ± standard deviation or median (range) and the between-the-groups comparisons are carried out using the Independent Samples T-test, or Mann-Whitney test, respectively. Statistical significance was set to .05

*FRP–*fluctuation-related pain, *LEDD*–Levodopa Equivalent Daily Dose, *SCOPA-Motor*–Scales for Outcomes in Parkinson’s Disease–Motor Function, *KPPS*–King’s Parkinson’s Disease Pain Scale, *PDQ-8* –Parkinson’s Disease Questionnaire–Short Form.

**Fig 2:**
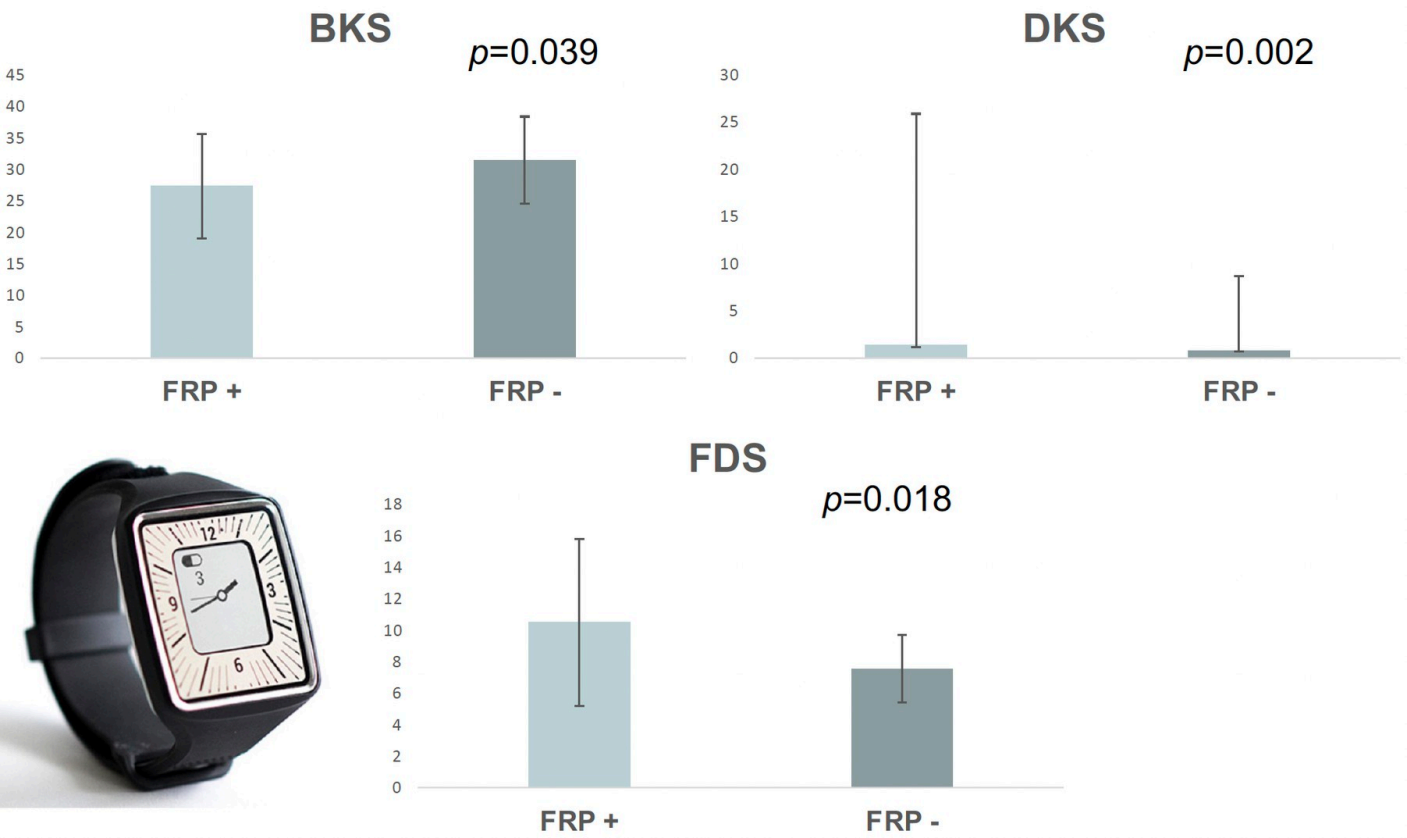
Comparison of Parkinson’s KinetiGraph™ (the PKG^TM^, Global Kinetics Corporation) scores between the groups of the participants with and without fluctuation-related pain (FRP). *BKS*—Bradykinesia Score, *DKS*—Dyskinesia Score, *FDS–*Fluctuation and Dyskinesia Score. Reprinted from https://pkgcare.com/wp-content/uploads/2021/09/Whitepaper_The-Long-Term-Leader-in-Parkinsons-Disease-Enhancing-your-Clinical-Trial-Assessment-MM-96.pdf under a CC BY license, with permission, original copyright 2021.

PwP who declared FRP had significantly lower BKS values (27.34±8.31 vs. 31.47±6.93, *p* = 0.039), higher DKS values (1.4 (0.2–24.5) vs. 0.8 (0.1–7.9), *p* = 0.002) and higher FDS values (10.50±5.32 vs. 7.56±2.16, *p* = 0.018), compared to participants without FRP (n = 40) Fig 2. In PwP with FRP, but not in those without, mean FDS was above the cut-off value proposed to distinguish fluctuators from non-fluctuators (FDS 7.7). (31) There was no statistically significant difference between the mean PTI (*p* = 0.314) and mean PTT scores (*p* = 0.684) in the two groups.

Next, clinical characteristics and the PKG^TM^-derived scores which differed significantly between participants with FRP and participants without FRP were probed as explanatory variables for the presence of FRP in a set of univariate logistic regression models. Here, three different PKG^TM^-obtained scores: BKS, DKS and FDS and four different clinical features: disease duration, H&Y stage, SCOPA-Motor total score and the presence of dyskinesia (SCOPA Motor, Item 20) were significantly impacting the presence of FRP Table 3.

**Table 3:** Results of univariate logistic regression.

Univariate logistic regression			
	Exp (B)	95% CI for Exp (B)	*p*
Fluctuation-related pain			
**Disease duration**	**1.139**	**1.024–1.267**	0.017*
Age	1.003	0.952–1.057	0.906
Gender	0.867	0.307–2.450	0.787
LEDD	1.001	1.000–1.002	0.111
**Hoehn & Yahr**	**1.904**	**1.095–3.312**	0.023*
**SCOPA-Motor total score**	**1.087**	**1.024–1.154**	0.006*
**SCOPA-Motor presence of dyskinesia**	**19.185**	**4.527–81.310**	<0.001*
SCOPA-Motor presence of “off” periods	2.794	0.944–8.266	0.063
**BKS**	**0.927**	**0.860–0.998**	0.045*
**DKS**	**1.323**	**1.047–1.671**	0.019*
**FDS**	**1.349**	**1.079–1.687**	0.009*
PTT	0.999	0.958–1.042	0.970
PTI	0.966	0.902–1.034	0.314

Statistical significance was set to .05

*CI*–confidence interval, *LEDD*–Levodopa Equivalent Daily Dose, *BKS*—Bradykinesia Score, *DKS*—Dyskinesia Score, *FDS–*Fluctuation and Dyskinesia Score, *PTT*–Percentage of Time with Tremor, *PTI*–Percentage of Time Immobile

**Fig 3:**
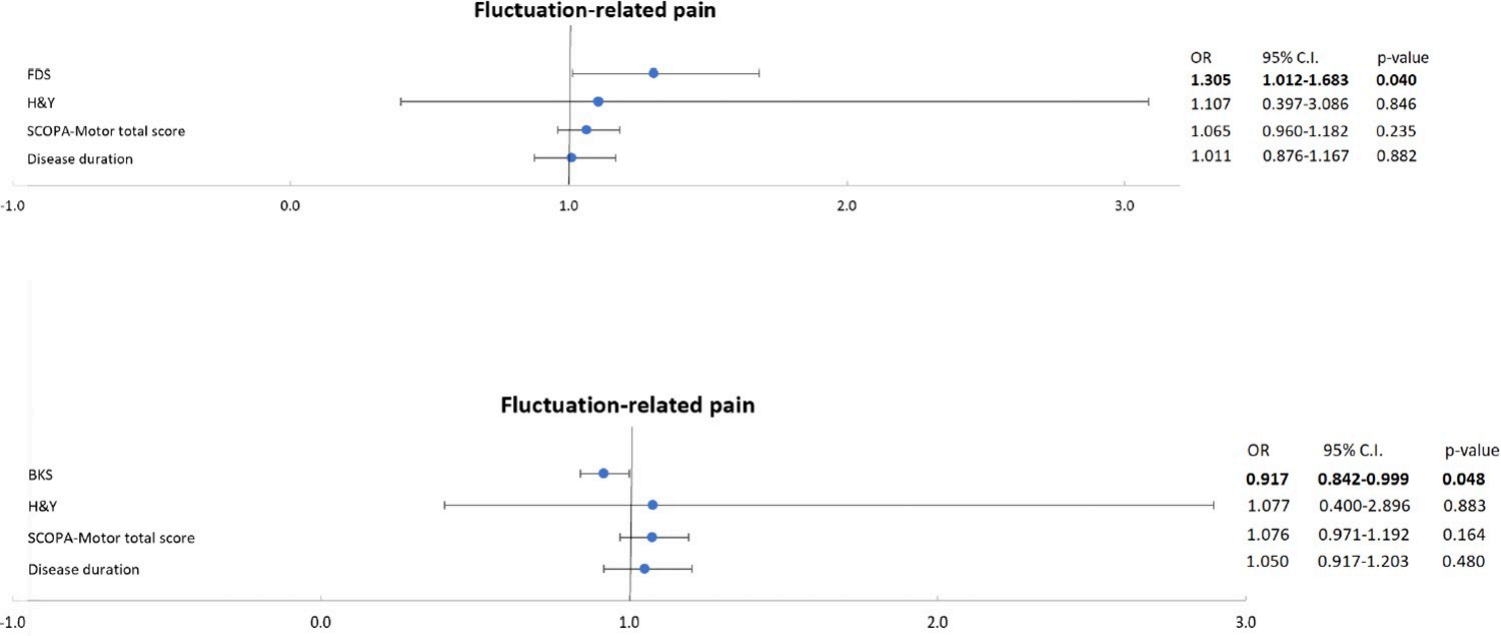
Multivariate logistic regression analysis identified a) the Fluctuation-Dyskinesia score (*FDS*) and b) the Bradykinesia Score (*BKS*) as significant predictors of fluctuation-related pain in People with Parkinson’s after the adjustment for Hoehn and Yahr Stage (*H&Y*) and the Scales for Outcomes in Parkinson’s disease–Motor Function (*SCOPA-Motor*) total score.

Finally, we built three distinct multivariate logistic regression models to probe the relationship between the three PKG^TM^ scores that significantly impacted FRP in the univariate regression analysis and the FRP (as a binary outcome) after controlling for clinical features significantly associated with FRP in the univariate regression analysis: disease duration, H&Y stage and SCOPA-Motor total score (which includes the score indicating the presence of dyskinesia, SCOPA-Motor Item 18). In the covariate-adjusted multivariate regression models, a significant relationship emerged between the FDS score and the presence of FRP (Exp (B) = 1.305, 95% CI for Exp (B) 1.012–1.683, *p* = 0.040; Fig 3A), as well as the BKS score and the presence of FRP (Exp (B) = 0.917, 95% CI for Exp (B) 0.842–0.999, *p* = 0.048; Fig 3B).

## Discussion

This explorative analysis evaluated potential links between the PKG^TM^ system-derived, accelerometer-based objective motor scores, and the presence of FRP in individuals with PD. Our key findings are:

- Two of the PKG^TM^-obtained objective motor outcome measures, namely the FDS score and the BKS score, were significantly associated with the presence of FRP following the adjustments for major clinical covariates—disease duration, H&Y stage, and the burden of motor symptom, including motor fluctuations.

In addition, we have found that:

- PwP suffering from FRP have longer disease duration and higher global motor disability, as well as higher presence of dyskinesia, then PwP without FRP.
- Health-related quality of life is worse in PwP with FRP compared to those without.

The role of DHT as tools for the assessment of PD-related pain in a home environment has not previously been investigated. However, in PD-free individuals, evidence of promising correlations between signals derived using such techniques and self-reported pain scores is emerging, some of which could possibly be translated into PD [46]. These pain assessment techniques rely on the evaluation of facial expressions, body movements, various physiological indicators or a fusion of all of the above [47]. Detailed discussion on those techniques is beyond the scope of this manuscript. Several examples include, but are not limited to, bed-based non-contact sensor monitoring used in palliative care; smartphone applications coupled with wearable devices with integrated sensors for electrodermal activity, bioimpedance, trunk motion-capture; wristbands with incorporated heart rate monitor, galvanic skin response and skin temperature sensor, three-axis accelerometer, and three-axis gyroscope; forehead wearable sensors for cerebral optical spectrometry and inertial measurement units, comprising both accelerometer and gyro sensor [47–54]. Among other techniques, wearable accelerometers measure aspects of health and behavior that may be directly related to pain and thus have clear value in the study and management of pain [46]. Nevertheless, pain, defined by the IASP as “an unpleasant sensory and emotional experience associated with or resembling that associated with actual or potential tissue damage” occurs in a biopsychosocial context and, as such, physiological measures cannot accurately denote the intensity of pain an individual experiences, but may merely indicate its presence or absence [24, 55].

Reporting of PD-related pain at clinical appointments is poor. In an online survey, only 10% of the total number of 115 participating PD patients (recruited via Parkinson’s UK Research Support Network) disclosed that they have discussed the pain they are experiencing with their neurologists, while in a cross-sectional analysis using the German Pain Questionnaire (181 enrolled participants with PD, 38.2% female, mean age 67.0±8.3 years), neurologists were involved in the pain management in only 3.3% participants [8, 56]. Moreover, as pain is an utterly unique, individual experience, one-point-in-time pain assessments, relying on self-reported pain scales or self-administered questionnaires collected during clinical appointments are often inadequate and may fail to capture the impact of pain on the individual’s function [46, 57]. Similarly, it remains difficult to accurately identify fluctuations of motor and NMS during a single in-person hospital visit–traditionally used clinical assessment tools are skewed by their retrospectivity, subjectivity and recall bias and can only provide an estimate [23, 58]. Embracing the DHT in the routine clinical care pathways would allow for measurements made in real-life environments, and for extending the duration of measurement over multiple days, enhancing its reliability [27, 46]. In a substantial proportion of PwP, this additional data collected between the clinical appointments may lead to changes, refinement and adjustment of the treatment plans, resulting in a greater improvement of the global motor and nonmotor disability (measured using the Movement Disorders Society-sponsored Revision of the Unified Parkinson’s Disease Rating Scale, *MDS-UPDRS total score*), compared to the management plans based solely on the traditional clinical assessment (75 PwP, mean H&Y 1.9 ± 0.6 vs. 79 PwP, mean H&Y 2.0 ± 0.6, respectively) [59, 60]. Importantly, in a viewpoint on their aspirations for future developments of digital health technologies, PwP themselves stressed that it should work for both motor and NMS [61].

Our findings indicate that signals derived from the PKG^TM^ may be associated with clinical ratings for FRP in PwP. Of note, while higher FDS scores increase, higher BKS scores decrease the odds for the presence of the FRP. These, seemingly contradictory, findings may possibly be explained by the previously reported reduction of nonmotor fluctuations amplitude (difference of symptoms severity between ON and OFF states) at late stages of PD. Response oscillations may occur as early as during the first year following the levodopa initiation, and, initially, increase in prevalence over the disease course [16]. However, a metanalysis of two cross-sectional studies with 101 PwP (45% female; median age: 71 (Interquartile Range, IQR, 65–78) years, median H&Y 3 (IQR 2.0–4.0) demonstrated that, although there is a clear increase of nonmotor burden with disease progression (defined based on H&Y stages), at late stages of PD the fluctuation amplitudes of both motor and NMS subside (mainly due to a greater increase of NMS severity in ON state compared to OFF state and an overall decrease in levodopa effects) [62]. PwP affected by FRP have longer disease duration and higher global motor disability, and it is possible that, in this group, higher severity of bradykinesia signposts further increases in symptom severity, when NMF, including FRP, may become less present, and other subtypes of PD-related pain may prevail.

Similarly to our study, previous studies emphasized the detrimental impact of FRP on health-related quality of life, as, for example, shown in the post-hoc analysis of the KPPS validation study including 178 PwP and 83 matched controls (measured using both the Parkinson’s Disease Questionnaire–Short Form, *PDQ-*8, and EuroQoL-5 Dimensions- 3 Levels, *EQ-5D-3L*) [10]. In an interview-based study (with participants recruited during their assessments at the Research Unit, Imperial College London, UK), when asked to rank their most troublesome symptoms in the past 6-month period, both PwP at the early (<6 years from the symptom onset, n = 92) and later (≥6 years from the symptom onset, n = 173) stages of the disease highlighted pain among the top-10 most troublesome symptoms they were experiencing [19]. Clearly, recognition, assessment, and management of PD-related pain in the clinic is of paramount importance. Especially in the context of FRP, this may be of a great value, as, in a subset of patients, this pain subtype can be ameliorated with adjustments of dopaminergic medication, ultimately raising the HRQoL [63–65].

Of note, our study revealed that FRP predominantly affects PwP at the advanced stages of the disease, and previous research pointed out reduced likeliness of this particular patient group to attend in person appointments with health-care professionals [66]. Moreover, patients with advanced disease may be affected by cognitive impairment and thus less capable of accurate self-reporting [46]. The PKG^TM^, which may, as demonstrated in the present study, flag up the presence of FRP in the home-based environment, may be helpful in lessening disability-related barriers and enhancing access to pain treatment, particularly for home-bound PwP.

Our study has certain limitations. Firstly, we present an exploratory, cross-sectional analysis of two independent studies that were not initially designed to address our research question, which could be more accurately investigated in a randomized controlled trial. Secondly, the findings of our cross-sectional analysis require replication in longitudinal trials, where an association between PKG^TM^-obtained scores and clinical pain ratings could be assessed in a long term. Due to the exploratory, pilot nature of our study, its sample size is relatively small and did not allow for separate analyses of each of the three distinctive items within the KPPS domain dedicated to FRP. Furthermore, while the KPPS inquiries about the PD-related pain present over the past month, the PKG^TM^-derived measurements are captured over 6 consecutive days. While we cannot exclude that this discordance may have led to an inconsistence in the assessed outcomes, it is in keeping with the use of wearables in previous clinical studies in PwP and in real-life clinical practice [26, 67]. Moreover, the lack of congruence between the time-period of pain presence assessed by the KPPS (one month) and the International Classification of Diseases, Eleventh Revision (ICD-11) definition of chronic pain as pain that persists or recurs for longer than 3 months may present an additional limitation [68]. However, the KPPS remains the most used assessment tool for PD-related pain in clinical trials globally and The International Parkinson and Movement Disorders Society (MDS), recommends its use for the assessment of pain intensity in PwP and suggests it for the syndromic classification of PD-related pain [36–38, 63, 69–79]. Despite its limitations, this is the first study to provide an insight into potential role of DHTs for the assessment of PD-related pain, and we feel that our findings (pending confirmation in larger trials) hold a potential to facilitate the recognition of FRP pain in PwP in clinical practice. Precise clinical phenotyping of PD-related pain based on the use of validated clinical assessment tools in a well-defined population of PwP additionally sets our study apart.

To conclude, our findings indicate that objective motor scores collected continuously in the home-based environment via the PKG^TM^ system are associated with clinical ratings for FRP obtained using validated clinical assessment tools. The PKG^TM^ system may thus advance the way we screen for, assess, and, consequently, treat FRP in the clinical practice. The findings of our small, explorative analysis necessitate larger, randomized controlled trials in enriched populations to estimate whether objective motor scores derived from the PKG^TM^ system may serve as a reliable and reproducible tool for pain management and research in the home setting, and whether the changes in the measured scores relate to the changes in the outcome. In the meantime, the PKG^TM^-obtained outcome measures may prompt clinicians to enquire about and address FRP, a frequently neglected symptom, at the clinical appointments.
